# A Comparative Study of Radiation Dose From Chest CT Scan Examinations in Two Imaging Centers in Duhok Province, Kurdistan, Iraq

**DOI:** 10.7759/cureus.75135

**Published:** 2024-12-05

**Authors:** Adil M Mahmoud

**Affiliations:** 1 Radiology, Azadi Teaching Hospital, Duhok, IRQ; 2 Clinical Radiology, College of Medicine, University of Duhok, Duhok, IRQ

**Keywords:** chest, ct scan, drls, ionizing radiation, radiation dose

## Abstract

Background

CT is among the most widely used diagnostic imaging techniques worldwide, providing significant advantages and invaluable diagnostic insights for detecting a wide range of diseases across various organs. However, it involves exposing patients to relatively high levels of ionizing radiation.

Objective

This study aims to document the radiation doses from chest CT scans performed at Azadi Teaching Hospital in Duhok Province and compare them with those recorded at the 3-Tesla Center for Advanced MRI and CT Scanning, also located in Duhok, using diagnostic reference levels (DRLs) as a benchmark.

Materials and methods

Data were gathered from the CT scanners and their data management systems at both Azadi Teaching Hospital and the 3-Tesla Center for Advanced MRI and CT Scanning. The study included daily records of unenhanced chest CT scans for 200 patients, with 100 scans from each facility. Data analysis was performed using IBM SPSS Statistics for Windows, Version 27.0 (Released 2020; IBM Corp., Armonk, NY, USA), and the DRLs were compared between the two centers.

Results

The gender distribution was nearly equal across both facilities, with most patients aged between 61 and 70. The mean volume CT dose index (CTDIvol) for chest CT scans was 279.39 mGy at Azadi Teaching Hospital and 227.14 mGy at the 3-Tesla Center. The mean dose length product (DLP) values were 655.14 mGy·cm and 789.61 mGy·cm, respectively, while the mean effective dose (ED) values were 9.171 mSv at Azadi Teaching Hospital and 11.054 mSv at the 3-Tesla Center. Interestingly, although the mean DLP and ED values were lower at Azadi Teaching Hospital compared to the 3-Tesla Center, the CTDIvol values did not show a statistically significant difference.

Conclusions

This study highlights the disparities in DRLs for chest CT scans between two medical institutions in Duhok Province. Higher mAs, DLP, and ED values were observed in some cases, suggesting that adult CT scanning protocols in Duhok may benefit from dose optimization strategies. Analyzing the impact of scanning parameters on dose descriptors and patient exposure, along with their effects on image quality, will help achieve the optimal balance for accurate diagnoses. Moreover, further research is needed to explore additional opportunities for dose optimization in this context.

## Introduction

CT scans are essential for modern medical diagnostics, providing high-resolution images of internal organs. However, it is crucial to balance the diagnostic benefits of CT imaging with the associated risks of radiation exposure, especially for patients who undergo frequent scans [1,2]. While CT scans represent a small proportion of all X-ray procedures, they account for a significant share of medical radiation exposure - up to 66% in the United States and 47% in the United Kingdom. Consequently, minimizing unnecessary CT scans and implementing strategies to shield patients from harmful radiation exposure are key priorities [3-5]. Optimizing CT scan parameters across radiology centers is a critical first step in achieving this goal. A key component of this optimization involves comparing CT parameters and patient radiation doses with diagnostic reference levels (DRLs) [6]. Radiology professionals use national DRLs as benchmarks to evaluate radiation dose metrics, ensuring compliance within the established ranges. When patient radiation doses exceed national DRLs, a comprehensive review of CT protocols is necessary, followed by adjustments to mitigate factors contributing to elevated radiation doses [7]. This issue is especially pertinent in regions such as Duhok Province in the Kurdistan region of Iraq, where healthcare professionals seek to enhance diagnostic accuracy while minimizing radiation exposure.

Azadi Teaching Hospital (a public facility) and the 3-Tesla Center for Advanced MRI and CT Scanning (a private center) are two distinct healthcare institutions in Duhok Province. While both provide vital imaging services, differences may exist in equipment, protocols, and the radiation doses administered during CT scans. This study aims to conduct a comparative analysis of the radiation doses patients receive during chest CT scans at Azadi Teaching Hospital and the 3-Tesla Center for Advanced MRI and CT Scanning. By examining and comparing radiation doses, imaging protocols, and equipment specifications, the study aims to identify potential differences in dose optimization strategies between these two healthcare facilities.

The findings of this study are intended to contribute valuable insights to the ongoing discourse on radiation safety in medical imaging, particularly in resource-limited regions. By identifying best practices and opportunities for improvement, the study’s conclusions could help guide decision-making processes aimed at enhancing patient safety and improving healthcare delivery in Duhok Province, as well as in similar settings across Iraq and globally.

## Materials and methods

Multi-detector, 64-slice Philips CT scanners (Koninklijke Philips N.V., Amsterdam, Netherlands) were used for all exams at both Azadi Hospital and the 3-Tesla Center. The chest scans were conducted following the manufacturer’s preset protocols in all instances. The scanning procedure covered the thoracic entrance level to the diaphragm and was performed after full inspiration. Key scanning parameters included a tube voltage of 120 kV, a tube current ranging from 250 to 450 mA, a slice thickness of 10 mm, and a slice spacing of 5 mm. After scanning, automated reconstruction generated thin-slice images with a thickness and spacing of 1.25 mm, which were stored as DICOM data. The reconstruction algorithm used was the lung algorithm, with a field of view of 500 mm × 500 mm and a matrix size of 512 × 512 pixels. In addition to the axial reconstructions, coronal and sagittal reconstructions were also available for all cases. The rotation time for the scanning procedure was set at 0.5 seconds, with the scan length ranging from 60 to 1,300 mm.

Data such as milliamperes-seconds (mAs), which measure the radiation output (milliamperage) over a set period (seconds), were obtained from the CT scanner via the X-ray tube. Other parameters recorded included the length of the scanned area (mm), scanning time (seconds), CT dose index volume (CTDIvol) in milligrays (mGy), and the dose length product (DLP) in milligrays per centimeter (mGy·cm) [8]. Additionally, the effective dose (ED), measured in millisieverts (mSv), was calculated using the formula: ED = DLP x k, where (k) is the tissue weighting factor for the scanned region (the chest, calculated at 0.014). This k factor was defined and endorsed by the International Commission on Radiation Protection (ICRP) in publications 60 and 103 [9-11].

Data were collected from the daily records of the CT scanners for single-phase, unenhanced chest scans of 200 patients (100 cases from each facility). CT scans with multiple phases were excluded from the analysis. The data were then analyzed using IBM SPSS Statistics for Windows, Version 27.0 (Released 2020; IBM Corp., Armonk, NY, USA), and DRLs were compared between the two healthcare centers.

Approval for this retrospective study was obtained from the ethics and scientific committee of the College of Medicine at the University of Duhok, Duhok Province, Kurdistan, Iraq. The committee also waived the requirement for informed consent.

## Results

A total of 200 single-phase non-enhanced chest CT scans were conducted, with 100 scans at each facility. The scans were performed on 98 males (43 at Azadi Hospital, 43.9%, and 55 at the 3-Tesla Center, 56.1%) and 102 females (57 at Azadi Hospital, 55.9%, and 45 at the 3-Tesla Center, 44.1%). There was no statistically significant difference in gender distribution between the two facilities (P = 0.09) (Table 1). 

**Table 1:** Gender distribution at Azadi Hospital and 3-Tesla Center

Gender	Azadi Hospital		3-Tesla Center		Total	
	Number	Percentage	Number	Percentage	Number	Percentage
Male	43	43.90%	55	56.10%	98	100%
Female	57	55.90%	45	44.10%	102	100%
Total	100	50%	100	50%	200	100%

**Figure 1:**
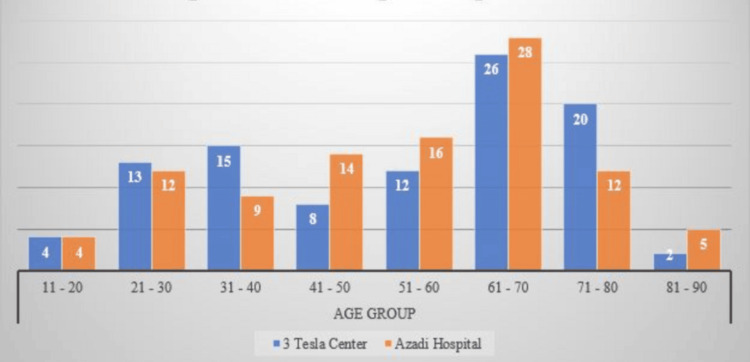
Age distribution per hospital site

Regarding patient age, the largest age groups at both sites were in the 61-70 age bracket, comprising 26% of patients at Azadi Hospital and 28% at the 3-Tesla Center. Four patients in the 11-20 age group were scanned at both institutions. Notably, however, the number of patients in the 21-30, 31-40, and 71-80 age brackets was higher at the 3-Tesla Center, with one, six, and eight more cases, respectively. In contrast, Azadi Hospital had higher numbers of patients in the 41-50, 51-60, and 81-90 age brackets, with increases of six, four, and three cases, respectively (Figure 1).

Additionally, all cases (n = 100) at Azadi Hospital included high-resolution CT (HRCT) scans, whereas none of the cases at the 3-Tesla Center underwent this procedure (Table 2).

**Table 2:** HRCT scans at Azadi Hospital and 3-Tesla Center HRCT, high-resolution CT

Hospital site	HR scan included?		Total
	Yes	No	
Azadi Hospital	100	0	100
3-Tesla Center	0	100	100
Total	100	100	200

Regarding the mAs readings at both institutes, a statistically significant difference was observed between the two sites (P < 0.001; 95% CI of -1.710 to 1.092). The mean mAs recorded at Azadi Hospital was 241.50, with an SD of 18.876 and an SEM of 1.888. In contrast, the 3-Tesla Center reported a mean of 287.04, with an SD of 41.888 and an SEM of 4.189 (Table 3).

**Table 3:** mAs levels recorded at Azadi Hospital and 3-Tesla Center

Hospital site	N	Mean	SD	SEM
Azadi Hospital	100	241.5	18.876	1.888
3-Tesla Center	100	287.04	41.888	4.189

The mean length of the scanned area (mm) was 335.3 mm at Azadi Hospital and 338.91 mm at the 3-Tesla Center, with SDs and SEMs of 36.627/3.973 and 47.603/4.607, respectively. There were no statistically significant differences between the sites (P = 0.584; 95% CI of -0.370 to 0.195) (Table 4).

**Table 4:** Length of the scanned area (mm) at Azadi Hospital and 3-Tesla Center

Hospital site	N	Mean	SD	SEM
Azadi Hospital	100	335.3	39.627	3.963
3-Tesla Center	100	338.91	47.603	4.76

Furthermore, the scanning time (seconds) was not significantly different between the two institutions (P = 1.302; 95% CI of -0.462 to 0.092). The mean scanning times were 5.3098 seconds at Azadi Hospital and 9.7542 seconds at the 3-Tesla Center, with SDs of 0.6973 and 34.1192, respectively, and SEMs of 0.0697 and 3.4119 (Table 5).

**Table 5:** Scanning times (seconds) at Azadi Hospital and 3-Tesla Center

Hospital site	N	Mean	SD	SEM
Azadi Hospital	100	5.3098	0.69733	0.06973
3-Tesla Center	100	9.7542	34.11924	3.41192

Regarding CTDIvol, there was no statistically significant difference between the two sites (P = 0.85; 95% CI of -0.7251 to -0.304). The Azadi Hospital CTDI records showed a mean of 279.39, with an SD of 1852.94 and an SEM of 185.294. The 3-Tesla Center CTDIvol records showed values of 227.14, 2052.17, and 205.217, respectively (Table 6).

**Table 6:** CTDI volume (mGy) at Azadi Hospital and 3-Tesla Center

Hospital site	N	Mean	SD	SEM
Azadi Hospital	100	279.3933	1852.946	185.2946
3-Tesla Center	100	227.1459	2052.177	205.2177

The mean, SD, and SEM of the DLP were 655.14, 80.775, and 8.0776 at Azadi Hospital. In comparison, the 3-Tesla Center showed values of 789.61, 131.593, and 13.1594, respectively (Table 7). Statistically significant differences were observed between the two sites (P < 0.001; 95% CI of -1.533 to 0.928).

**Table 7:** Total DLP (mGy.cm) values at Azadi Hospital and 3-Tesla Center DLP, dose length product

Hospital site	N	Mean	SD	SEM
Azadi Hospital	100	655.141	80.7759	8.0776
3-Tesla Center	100	789.618	131.5936	13.1594

The ED values were also significantly different between the two sites (P < 0.001; 95% CI of -1.533 to 0.928). The mean, SD, and SEM values for ED were 9.171, 1.138, and 0.1138 at Azadi Hospital, and 11.054, 1.8423, and 0.18423 at the 3-Tesla Center, respectively (Table 8).

**Table 8:** ED (DLP × k) values at Azadi Hospital and 3-Tesla Center ED, effective dose

Hospital site	N	Mean	SD	SEM
Azadi Hospital	100	9.171974	1.130862	0.113086
3-Tesla Center	100	11.05465	1.842311	0.184231

## Discussion

This study measured and compared the radiation dose parameters for unenhanced single-phase adult chest CT scans at Azadi Teaching Hospital and the 3-Tesla Center for Advanced MRI and CT Scanning in Duhok Province, Kurdistan. This research fills a gap in the scientific literature, as the topic has not been extensively studied in this region. The findings are expected to contribute valuable insights into radiation dose management and its implications for patient safety within local and regional healthcare settings.

The DRL, as defined by the ICRP in its latest publication, Report 135, serves as an investigation level to optimize patient protection during medical procedures involving ionizing radiation. DRLs help determine the radiation dose for scanning specific organs or body parts [12] and set exposure limits to prevent overexposure. It is universally acknowledged that image quality must be sufficient to ensure accurate diagnosis, and reducing radiation dose should not compromise this quality. The As Low As Reasonably Achievable (ALARA) principle aims to minimize radiation exposure without sacrificing image quality. However, there are circumstances where increased radiation doses may be justified in specific cases [13].

Unenhanced chest CT scans are among the most common imaging procedures globally, typically used for patients with pulmonary symptoms such as shortness of breath or chest pain, as well as for screening those at risk for lung cancer [14]. Variations in CT dose parameters across different hospitals, as well as higher DLP values observed in certain cases, suggest that there are opportunities for optimizing radiation doses in chest CT examinations in Duhok Province. By analyzing the relationship between scan parameters, dose descriptors, patient exposure, and image quality, the study can contribute to achieving a balance that ensures both accurate diagnoses and minimal radiation exposure. Further research in this area is needed to explore additional optimization opportunities [15,16].

One factor that influences DLP is the scan length. While mA directly affects CTDIvol and, consequently, DLP, acceptable CTDIvol values indicate that appropriate mA settings are used. Longer scan lengths generally result in higher DLP values. The extent of the anatomy imaged during a CT scan is determined by clinical necessity, as longer scan lengths lead to increased radiation exposure. Elevated DLP values could suggest that certain regions, such as the chest, are being overscanned. However, since patient height or body size was not standardized in this study, variations in scan length could reflect individual physical characteristics rather than methodological issues [17,18].

Although both healthcare institutions used the same model of CT scanner (Philips 64-Slice), the radiation dose parameters at the 3-Tesla Center were significantly higher than those at Azadi Hospital, with statistically significant differences observed in the mAs, DLP, and ED values. However, no significant differences were found in scan length, scan time, or CTDIvol between the two sites. These differences may be attributed to the factory settings of each scanner. Additionally, factors such as improperly calibrated equipment, insufficient training of technical staff, and other potential contributors could explain these discrepancies.

The imaging protocols for chest CT scans also differed between the two sites. At Azadi Hospital, high-resolution acquisitions (HRCT) were routinely included as part of the protocol, whereas none of the patients at the 3-Tesla Center underwent HRCT chest scans. This variation is another factor that warrants attention and consideration by the healthcare personnel responsible for establishing and optimizing imaging protocols.

## Conclusions

This study highlights the disparities in DRLs for chest CT scans between two medical institutions in Duhok Province. Higher values for mAs, DLP, and ED were observed in certain cases, suggesting that adult CT scanning protocols in Duhok may benefit from optimized radiation dose strategies. Analyzing how scanning parameters influence dose descriptors, patient exposure, and image quality will help achieve the necessary balance for accurate diagnoses. Further research is needed to identify additional opportunities for dose optimization. Finally, the author recommends the implementation of periodic staff training programs and regular quality assurance inspections for the equipment to ensure optimal radiation dose management and patient safety.
